# Bismuth Plasmonic Antennas

**DOI:** 10.1021/acsnano.5c07482

**Published:** 2025-09-01

**Authors:** Michael Foltýn, Tomáš Šikola, Michal Horák

**Affiliations:** † 48274 Brno University of Technology, Central European Institute of Technology, Purkyňova 123, Brno 612 00, Czech Republic; ‡ Brno University of Technology, Faculty of Mechanical Engineering, Institute of Physical Engineering, Technická 2, Brno 616 69, Czech Republic

**Keywords:** bismuth, nanophotonics, plasmonic antennas, localized surface plasmons, electron energy loss spectroscopy

## Abstract

Bismuth is a particularly promising alternative plasmonic metal because of its theoretically predicted wide spectral bandwidth. In this study, we experimentally demonstrate the correlation between the shape and size of individual bismuth plasmonic antennas and their optical properties. To this end, we employ a combination of scanning transmission electron microscopy and electron energy loss spectroscopy. Bar-shaped and bowtie bismuth plasmonic antennas of various sizes were fabricated by focused ion beam lithography of a polycrystalline bismuth thin film. Our experimental findings demonstrate that these antennas support localized surface plasmon resonances and their dipole modes can be tuned through their size from the near-infrared to the entire visible spectral region. Furthermore, our findings demonstrate that bismuth exhibits a plasmon dispersion relation that is nearly identical to that of gold while maintaining its plasmonic performance even at higher plasmon energies, thus rendering it a promising low-cost alternative to gold.

## Graphical abstract

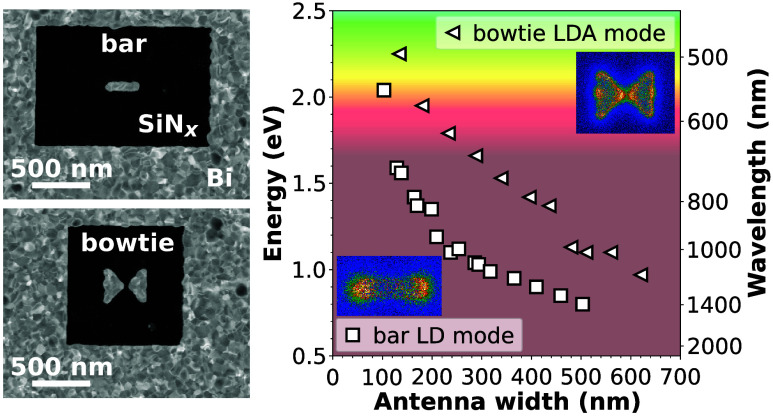

Collective oscillations of free electrons in metallic nanoparticles, called localized surface plasmon resonances (LSPR), are known to enhance the local electromagnetic field in the vicinity of nanoparticles.^1^ These extraordinary properties of plasmonic nanostructures, often called plasmonic antennas, have been used in various applications, including biosensing,^2,3^ catalysis,^4^ and ultrathin optical elements.^5,6^ Gold has been a material of choice and subject of study for many years in the context of plasmonic applications. However, the strong damping of LSPRs at energies above 2 eV, caused by the gold interband transitions, limits the use of gold plasmonic antennas to the near-infrared and a part of the visible spectral region.^7^ This limitation has prompted the exploration of alternative non-noble plasmonic metals, such as gallium,^8^ magnesium,^9^ potassium,^10^ aluminum,^11^ silver amalgam,^12^ and vanadium dioxide.^13,14^ Another material that has been theoretically predicted to offer a spectral interval wider than the visible region is bismuth.^15^

The low effective mass of free electrons and the dielectric function of bismuth suggest that it is an attractive plasmonic material suitable for plasmonics spanning from the near-infrared to the ultraviolet spectral region.^16,17^ Furthermore, the extraordinary properties of bismuth, including quantum confinement,^18^ temperature-induced metal-to-semiconductor transition,^19^ and high values of the Seebeck coefficient,^20^ when combined with its plasmonic performance, have the potential to yield new applications. Despite theoretical predictions of plasmonic activity in bismuth,^15^ experimental research in this field has been limited to investigating circular or spherical bismuth nanostructures using far-field optical spectroscopy.^21–25^ The primary challenge of the method is that it mostly measures the overall response of an ensemble of nanoparticles rather than of individual ones. This is because real samples generally contain nanoparticles of various sizes, resulting in plasmonic resonances that mutually overlap in the measured spectrum. Consequently, it becomes impossible to isolate the contribution of a single nanoparticle.^26,27^ As a result, the exploration of the spectral tunability of LSPRs in individual bismut nanostructures as a function of their size using previously employed techniques is rendered unfeasible.

In this work, we present a study that uses electron energy-loss spectroscopy in a scanning transmission electron microscope (STEM-EELS)^28^ to address the optical response of individual bismuth plasmonic antennas. We show the spectral tunability of the dipole LSPR modes over the near-infrared and visible spectral range and correlate it with the size of bismuth nanostructures.

## Results and Discussion

Bismuth plasmonic antennas were prepared using a standard focused ion beam (FIB) lithography process^29^ and characterized using STEM-EELS.^30^ The schematic workflow for the fabrication and characterization of bismuth plasmonic antennas is shown in Figure 1. Thirty nm thick bismuth films were deposited on commercially available silicon nitride membranes by magnetron sputtering (Figure 1a). The micrograph obtained by scanning electron microscopy (SEM) shows the deposited bismuth layer with polygonal grains. The cross-sectional view of a lamella cut off from the sample shows a pronounced roughness of the bismuth polycrystalline layer (see Figure S1). To further assess the oxidation resistance of the bismuth thin films when exposed to air, we employed a series of diffraction experiments while the diffractograms measured approximately half a year after deposition exhibited no indications of any bismuth oxide crystal phases (see Figure S2). Plasmonic antennas were fabricated from bismuth thin films by FIB lithography (Figure 1b). We targeted bar-shaped and bowtie bismuth plasmonic antennas. The width of the bar-shaped antennas is 80 nm and their length varies from 100 to 500 nm. Bowtie antennas have a wing angle of 90° and their total length, which is equal to their width, ranges from 130 to 620 nm. FIB lithography patterns with marked length of the bar-shaped antenna and width of the bowtie antenna are shown in Figure 1b together with STEM annular dark field (ADF) micrographs of 286 nm long bar and 288 nm wide bowtie antenna. Figure S3 shows the thickness maps and thickness profiles of a 410 nm long bar-shaped and a 288 nm wide bowtie bismuth antenna measured by STEM EELS. Figure 1c shows a setup of electron energy loss spectroscopy used to measure the EELS of individual bismuth nanostructures with analysis of the 294 nm long bar antenna. It includes a STEM ADF micrograph, a background-subtracted electron energy loss spectrum integrated over the left corner of the antenna with a peak at 1.0 eV corresponding to a longitudinal dipole LSPR mode, and the loss probability map at the peak energy (1.0 eV) showing the spatial distribution of this mode with two maxima at the corners of the antenna.

**Figure 1:**
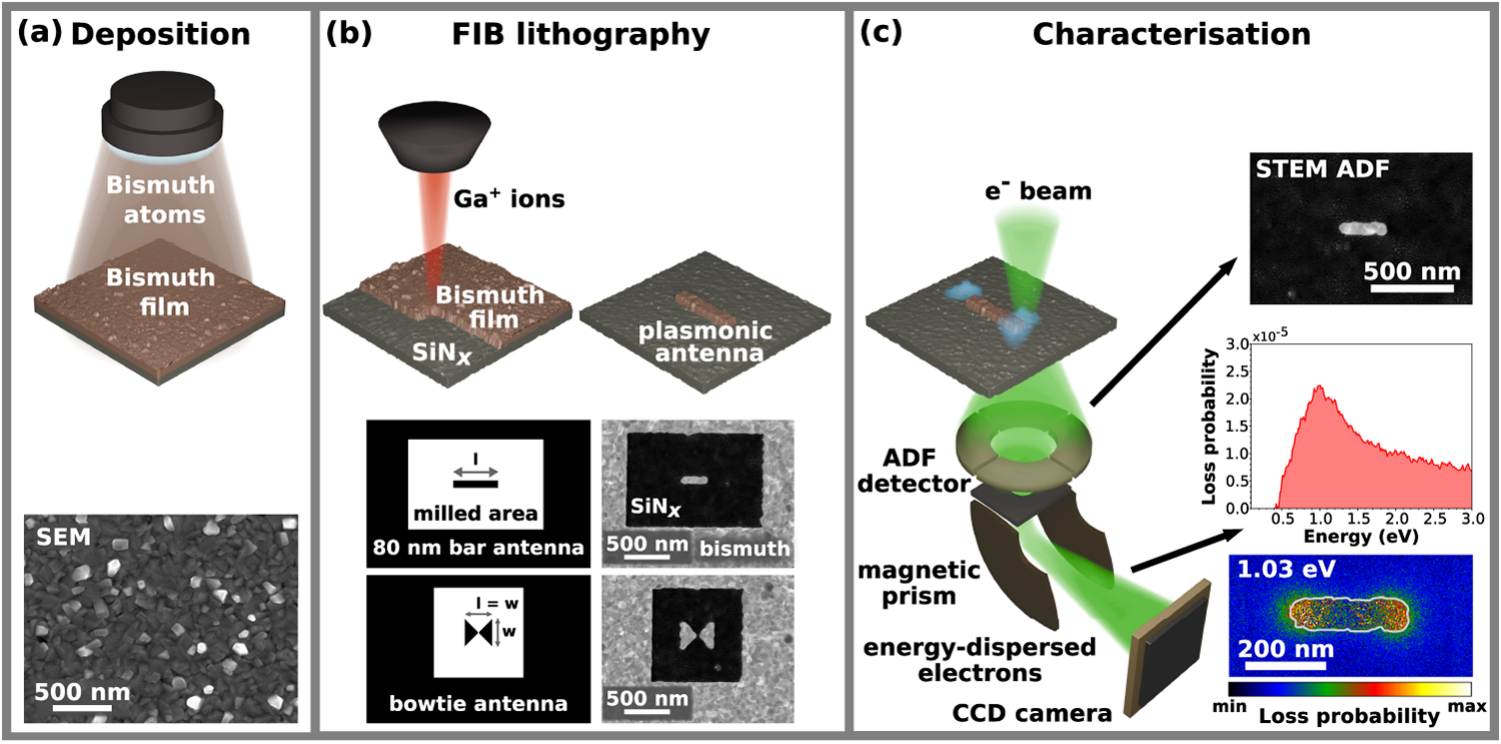
Schematic workflow for the fabrication and characterization of bismuth plasmonic antennas: (a) bismuth thin films were deposited by magnetron sputtering on a silicon nitride membrane, (b) bar-shaped and bowtie bismuth plasmonic antennas were fabricated by FIB lithography, and (c) their morphology was captured by STEM ADF micrographs and LSPRs were explored by STEM EELS.

First, we have inspected the set of 16 bar-shaped antennas with a length from 103 to 503 nm. The results are summarized in Figure 2. Figure 2a presents EEL spectra measured on the edges of fabricated bar antennas (the integration area is marked in Figure 2b by the black rectangle), whose STEM ADF micrographs are depicted in Figure 2c. These EEL spectra exhibit pronounced peaks in the energy region 0.8 to 3.2 eV corresponding to the longitudinal dipole (LD) and longitudinal quadrupole (LQ) LSPR mode, which are schematically shown in Figure 2b. The identification of modes was performed with the help of numerical simulations and loss probability maps. A comparison of the EELS experiment and the theory for the 400 nm bar is shown in Figure S4 and the loss probability maps of all bar antennas are shown in Figure S5. The measured EEL spectra were fitted by two Gaussian curves to extract the characteristic parameters of the observed individual plasmon peaks. These parameters are the peak position corresponding to the energy of the respective LSPR mode, the loss probability maximum, and the full width at half-maximum (FWHM) of the peak (see Table S1). The loss probability maxima obtained for both LD and LQ modes remain approximately the same for all antenna lengths, with an observable decrease for the shortest antennas.

**Figure 2:**
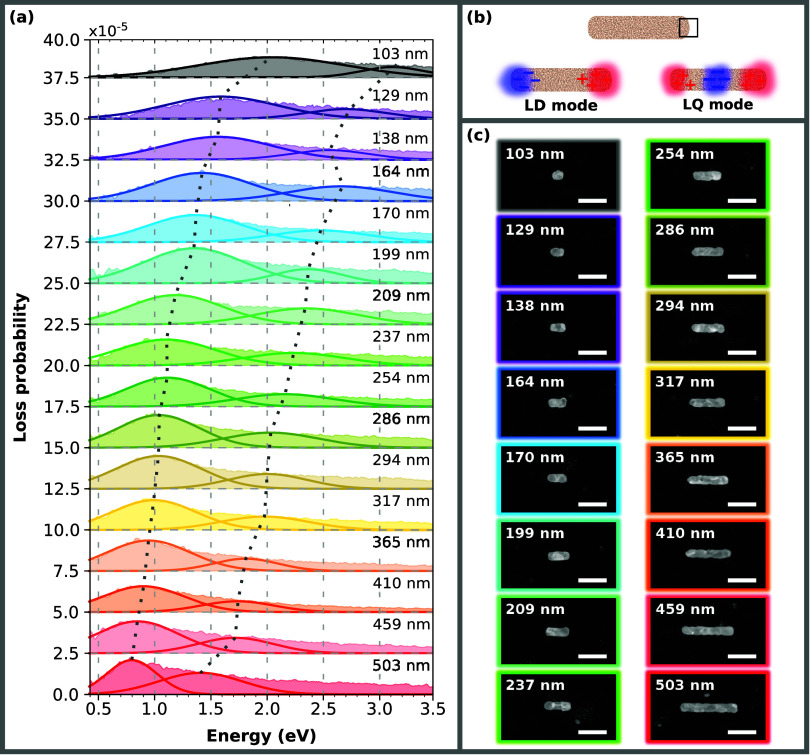
EELS analysis of bar antennas: (a) Measured EEL spectra (further fitted with two Gaussians) of bar antennas with the length ranging from 103 to 503 nm. The first peak in every spectrum corresponds to the longitudinal dipole (LD) mode and the second to the longitudinal quadrupole (LQ) mode. The dashed lines are guides for the eye and follow the energy of LD and LQ modes that increases with the decreasing length of the bar antenna. (b) A schematic of the LD and LQ mode with the marked area at the edge of the antenna where the EEL spectra were collected. (c) STEM ADF micrographs of individual analyzed bar antennas. The length of the scalebars is 250 nm.

In the following, we will focus on the LD mode. The highest loss probability of 2.3 × 10^–5^ was observed for the 199 nm antenna, while the lowest loss probability of 1.3 × 10^–5^ was observed for the 103 nm antenna. Similarly, the FWHM of fitted plasmon peaks remains roughly constant for most of the antennas, with an observable increase for the shortest antennas only. The lowest FWHM of 0.22 eV was observed in the 503 nm long antenna. For all remaining antennas longer than 240 nm, the FWHM remains below 0.38 eV. For shorter antennas, the FWHM increases to 0.65 eV for the shortest antenna. For bar antennas longer than 200 nm, the plasmonic response such as the peak intensity and the FWHM remains comparable, offering stable plasmonic performance regardless of the antenna dimension. Consequently, bismuth bar antennas represent a vivid plasmonic system tunable from the visible to the near-infrared spectral region.

Second, we have investigated the set of 11 bowtie antennas with a width from 134 to 620 nm. The results are summarized in Figure 3. To characterize the plasmonic performance, we studied the EEL spectra at the bowtie corners (Figure 3a) and at the gap between its two wings (Figure 3b). The integration area where the spectra were collected is depicted in Figure 3c together with a schematic of the transverse dipole (TD) mode with the maxima at the outer corners of the bowtie and the longitudinal dipole antibonding (LDA) mode with the maximum at the gap of the bowtie. The peaks in the measured EEL spectra and their respective plasmon modes were identified with the help of numerical simulations and loss probability maps. A comparison of the EELS experiment and the theory for the 288 nm bowtie is shown in Figure S6, and the loss probability maps of the TD and LDA modes of all bowtie antennas are shown in Figure S7. The peak energy, loss probability maxima, and FWHM of the TD and LDA modes were obtained by fitting the measured spectra with Gaussian curves (see Table S2). In the case of the TD mode, the loss probability is the highest for antennas with widths from 180 to 480 nm. The highest value of 2.6 × 10^–5^ is reached by the 396 nm bowtie. For bowties below and above the specified antenna width range, the loss probability decreases. The lowest observed loss probability of 1.1 × 10^–5^ was measured in the 480 nm antenna. The FWHM of the TD plasmon peaks increases with decreasing antenna width. The lowest FWHM of 0.17 eV was evaluated for the 620 nm bowtie, whereas the highest FWHM of 0.56 eV was observed for the 180 nm antenna. Antennas with widths between 233 and 480 nm exhibit TD plasmon peaks of comparable FWHM values between 0.30 and 0.36 eV. In the case of the LDA mode, the loss probability increases with the width of the antennas and culminates at 4.4 × 10^–5^ for the 396 nm bowtie. The lowest loss probability of 1.4 × 10^–5^ was measured for the smallest (134 nm) antenna. The FWHM generally increases with decreasing antenna width, from the lowest value of 0.25 eV of the largest antenna to 0.50 eV of the second smallest bowtie.

**Figure 3:**
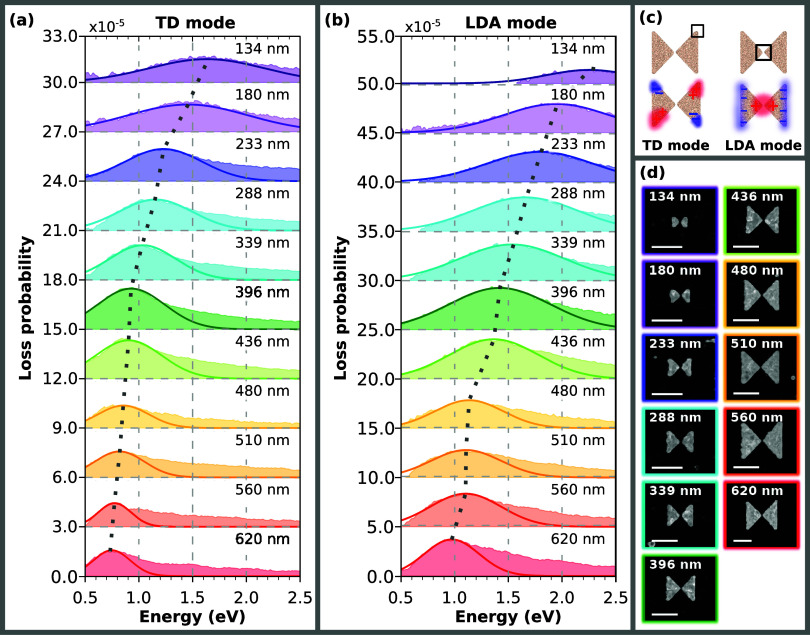
EELS analysis of bowtie antennas: (a,b) Measured EEL spectra (further fitted with a Gaussian) from the outer corners (a), where the peak corresponds to the transverse dipole (TD) mode, and gaps (b), where the peak corresponds to the longitudinal dipole antibonding (LDA) mode, of bowtie antennas with the width ranging from 134 to 620 nm. Dashed lines are guides for the eye and follow the energy of TD and LDA modes that increases with the decreasing width of the bowtie antenna. (c) Schematic depiction of the TD and LDA mode with marked areas where the EEL spectra were collected. (d) STEM ADF micrographs of the analyzed bowtie antennas. The length of the scalebars is 400 nm.

Third, we have evaluated the spectral tunability of bismuth plasmonic antennas. The plasmon resonances in both the bowtie and bar antennas range from the near-infrared to the visible part of the spectrum.

In the case of bar antennas (Figure 4a), the LD mode energy covers the interval from 0.80 eV (corresponding to 1550 nm in wavelength) for the longest 503 nm antenna to 2.04 eV (608 nm in wavelength) for the shortest 103 nm one. The LQ mode energy covers the energy interval from 1.41 eV (879 nm in wavelength) for the longest antenna to 3.13 eV (396 nm in wavelength) for the shortest one. The Q factor, defined as the LSPR energy divided by its FWHM, of the LD mode remains constant for all antenna lengths and fluctuates around the value of 3. In the case of the LQ mode, the Q factors are higher than those for the LD modes. For the shortest antenna, the Q factor of the LQ mode is the highest, reaching a value of 8.6. With increasing antenna width, the Q factor values decrease, reaching a minimum of 3.8 for the longest antenna.

**Figure 4:**
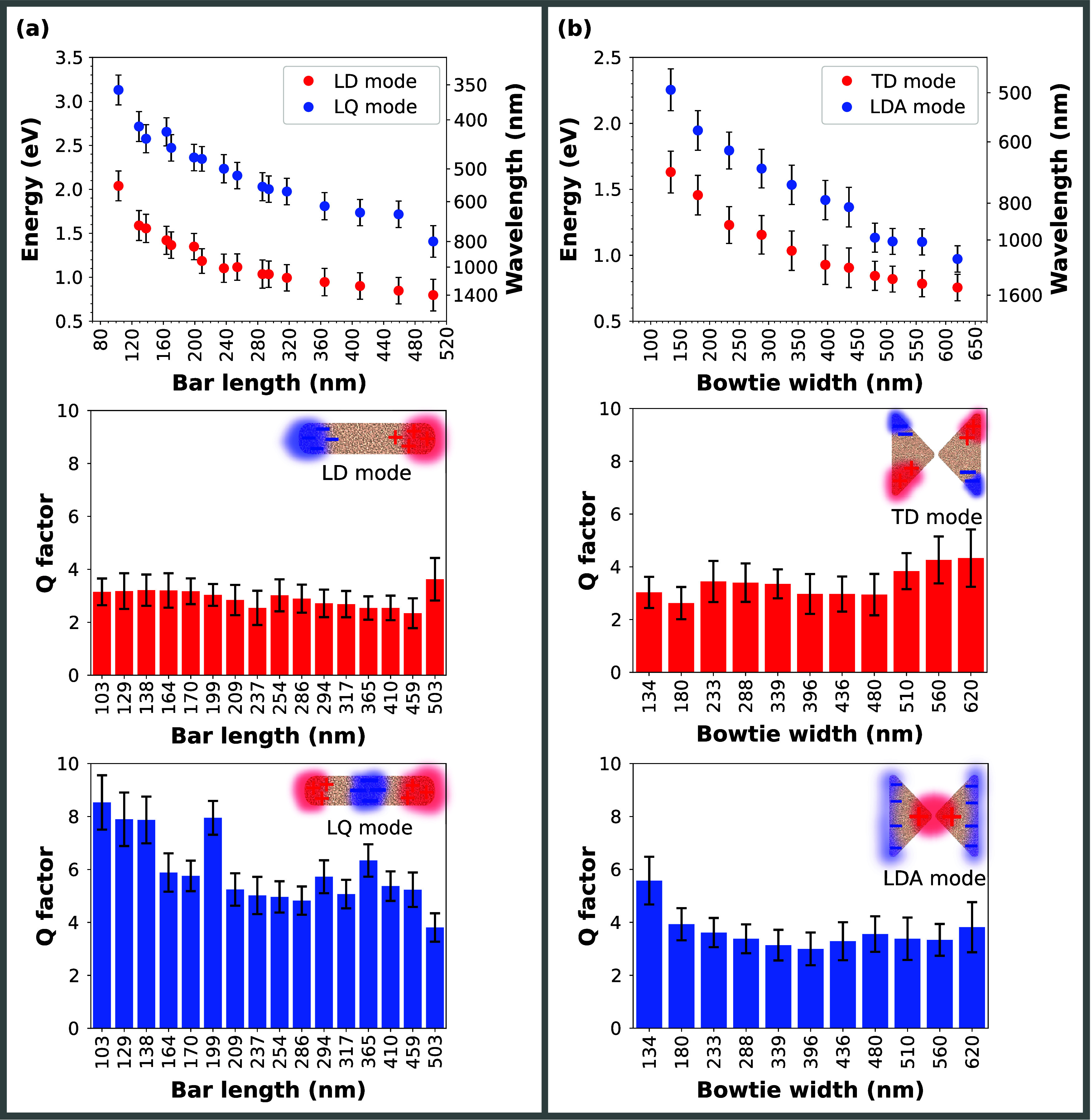
Spectral tunability of bismuth plasmonic antennas: (a) Plasmon energy and *Q* factors of the LD and LQ modes extracted from the measured EEL spectra shown in Figure 2 as a function of the length of the bar antennas. (b) Plasmon energy and Q factors of the TD and LDA modes extracted from the measured EEL spectra shown in Figure 3 as a function of the width of the bowtie antennas.

In the case of bowties (Figure 4b), the energy of the TD mode ranges from 0.76 eV (1631 nm in wavelength) for the largest 620 nm antenna to 1.63 eV (761 nm in wavelength) for the smallest 134 nm antenna. The LDA mode energy interval covers the interval from 0.97 eV (1278 nm in wavelength) for the largest antenna to 2.25 eV (551 nm in wavelength) for the smallest. The Q factors of the TD modes fluctuate around the value of 3 for all bowtie antenna widths, except for the largest ones for which the Q factor increases over 4. In the case of the LDA modes, the Q factors are higher and fluctuate constantly around the value of 3.5 except for the smallest antennas (widths around 200 nm and smaller), where the Q factors increase, reaching a maximum of 5.6 for the smallest bowtie antenna.

In summary, the dependence of the plasmon resonance energy on the antenna size proves the suitability of bismuth to cover the near-infrared spectral region and, with sufficiently small (below 100 nm) nanostructures, even the entire visible region while maintaining its plasmonic performance throughout the spectral bandwidth. However, the use of lithographically fabricated nanostructures of the two tested geometries is not capable of supporting LSPR in the ultraviolet spectral region, as very small structures (below 50 nm in size) would be necessary. Such small structures can be achieved by, for example, a bottom-up process such as chemical synthesis.

Finally, we compare the plasmonic properties of bismuth with those of gold. A direct comparison of the plasmon resonance energies of the TD and LDA modes in bismuth and gold bowties is shown in Figure 5. The pseudodispersion relations for these two modes in bismuth bowties overlap with the corresponding pseudodispersion relations for gold bowties. The observed overlap of the pseudodispersion relations suggests that bismuth can be considered as an alternative material to gold. The Q factors of the bismuth antennas are marginally lower than those of gold (see Figure S8), however, this disadvantage is counterbalanced by their consistent performance even at higher plasmon energies. Furthermore, the significantly lower cost of bismuth antennas enhances their applicability.

**Figure 5:**
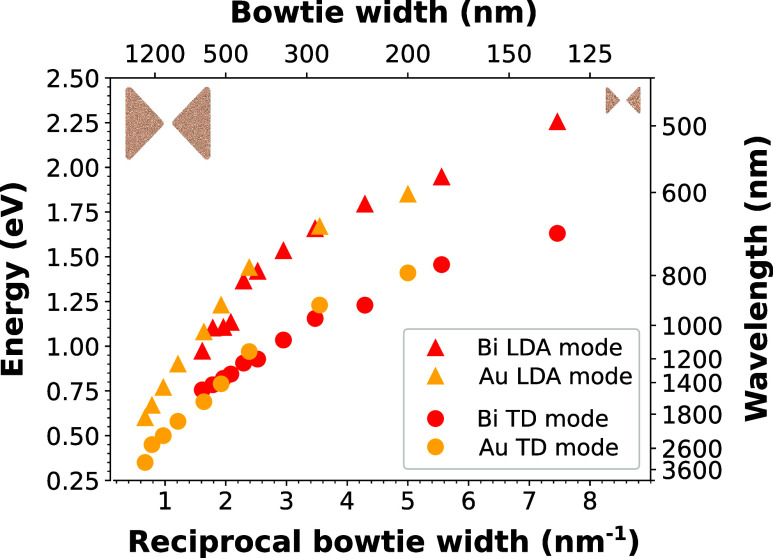
Pseudodispersion relation of LSPR in bismuth and gold bowties: Energy of the TD and LDA modes in bismuth (data from Figure 4b) and gold (data from ref 31) bowties is plotted as a function of the reciprocal antenna width. The overlap of the dependencies for both materials suggests the full substitutability of gold by bismuth in plasmonic applications.

It is imperative to consider two other pivotal factors that influence the suitability of various plasmonic materials: their biocompatibility and chemical stability under ambient conditions, such as their resistance to oxidation when exposed to air. In both cases, bismuth exhibited properties comparable to those of gold. The present study employed a series of diffraction experiments using both X-ray and electron diffraction to assess the oxidation resistance of bismuth thin films when exposed to air. The diffractograms measured approximately half a year after deposition exhibited no indications of any bismuth oxide crystal phases, despite prolonged exposure of the bismuth film to ambient conditions. Consequently, bismuth in the form of thin polycrystalline layers appears to be chemically stable and resistant to oxidation. However, bismuth can be easily oxidized in an oxygen-reactive atmosphere, such as oxygen plasma, or by annealing under an oxygen atmosphere. The diffractograms are included in Figure S2. As a result, bismuth can be easily covered by a few nanometers of oxide layer that may act as an insulator and, for example, prevent charge transfer in catalytic reactions. On the contrary, gold is resistant to oxidation under any conditions. Further, we note that a limiting factor in high-temperature applications might be the melting temperature of bismuth, which is much lower (271.5 °C) than the melting temperature of gold (1064 °C).

In terms of its toxicity, bismuth is generally considered biocompatible. The only known bodily harm caused by bismuth is inflammation of the lungs after inhalation of fine bismuth powder, which is caused by mechanical irritation of the tissue. However, when bismuth is introduced into the body in other forms, no negative effects on mammals have been observed, even at doses as high as 1000 mg per 1 kg of body mass.^32,33^ Consequently, bismuth emerges as a promising alternative to gold, offering cost-effectiveness, chemical stability, biocompatibility, and enhanced plasmon energy tunability, albeit at the expense of reduced plasmon resonance intensity.

## Conclusions

In conclusion, we have fabricated bar-shaped and bowtie bismuth plasmonic antennas using a standard focused ion beam lithography of a polycrystalline bismuth thin film and characterized them using STEM-EELS. This approach enables the study of plasmonic properties at the single-particle level and the exploration of the spectral tunability of localized surface plasmon resonances in individual bismuth nanostructures as a function of their size and shape. The spectral tunability of single modes over the near-infrared and visible spectral range has been demonstrated, and a correlation with the size and shape of bismuth nanostructures has been established.

Our experimental results show that bismuth is a suitable and cost-effective material for plasmonic applications. The dipole modes in the explored nanostructures are tunable from the near-infrared spectral region to the entire visible region. Furthermore, the plasmon resonances exhibited by these structures are found to be stable over the entire plasmon energy interval. Moreover, we have shown that the dependence of the plasmon energy on the antenna size for gold and bismuth is highly congruent, thereby establishing bismuth as a viable alternative to gold. This is further underscored by the observation that bismuth also covers the energies above 2 eV. In addition, the lower cost of bismuth, together with its biocompatibility and resistance to oxidation, make it a suitable candidate for use, especially in industrial and large-scale plasmonic applications.

## Methods

### Metal Deposition

A 30 nm thick bismuth thin film was deposited on a 30 nm silicon nitride membrane (by Agar Scientific) with lateral dimensions of 250 × 250 μm^2^ by DC magnetron sputtering. We used Magnetron Sputtering System BESTEC with the following parameters: chamber pressure 8·10^–4^ mbar, sample rotation 5 rpm, argon gas flux 15 sccm, argon ion energy 310 eV, and total current of argon ions 25 mA resulting in the deposition rate of 0.45 Å s^–1^.

### FIB Lithography

FIB lithography of the polycrystalline bismuth thin film was performed using FEI Helios by gallium ions with an energy of 30 keV and an ion beam current of 2 pA. We note that the highest available beam energy and the lowest available beam current are optimized for the best spatial resolution of the milling.

### EELS Measurement

EELS measurements were carried out in a TEM FEI Titan equipped with a GIF Quantum spectrometer operated at 120 keV in the scanning monochromated mode with the convergence semiangle set to 10 mrad and the collection semiangle set to 11.4 mrad. The probe current was adjusted to around 100 pA. The dispersion of the spectrometer was set to 0.01 eV per channel and the FWHM of the zero-loss peak was around 0.15 eV. The acquisition time was adjusted to use the maximal intensity range of the CCD camera in the spectrometer and avoid its overexposure. EEL spectra were integrated over rectangular areas at the edges of the nanostructures where the LSPR is significant. They were further divided by the integral intensity of the zero-loss peak to transform the measured counts into a quantity proportional to the loss probability (i.e., the zero-loss peak area equals 1), background subtracted by subtracting the EEL spectrum of a pure silicon nitride membrane (so the resulting spectrum starts at 0 intensity at the low-energy side), and fitted by Gaussians. We note that no deconvolution was applied.

### Numerical Simulations

Numerical simulations of EELS spectra were performed using the MNPBEM toolbox^34^ based on the boundary element method. The dielectric function of bismuth was taken from ref 35 and the surrounding dielectric constant was set to 1.6 to approximate the effect of the silicon nitride membrane substrate. The 120 keV electron beam was positioned 5 nm outside the antenna. The loss probability density was further recalculated to the loss probability at energy intervals of 0.01 eV corresponding to the dispersion of the spectrometer in the experiment.

## Supplementary Material



## Data Availability

Data sets for this manuscript are available in Zenodo at 10.5281/zenodo.15130647.

## Notes

EDX, SAED and XRD analysis of the bismuth thin film, thickness maps and thickness profiles of a bar-shaped and bowtie antenna, plasmonic response of a 410 nm long bar-shaped bismuth antenna (simulations and experiment), parameters of LD and LQ mode in the bar antennas, energy-filtered loss probability maps of the bar antennas, plasmonic response of a 288 nm wide bowtie bismuth antenna (simulations and experiment), energy-filtered loss probability maps of the bowtie antennas, parameters of TD and LDA mode in the bowtie antennas, and comparison of the Q factors for the TD and LDA modes in the bowtie bismuth and gold antennas (PDF)

## References

[1] Schuller J. A., Barnard E. S., Cai W., Jun Y. C., White J. S., Brongersma M. L. (2010). Plasmonics for extreme light concentration and manipulation. Nat. Mater..

[2] Klinghammer S., Uhlig T., Patrovsky F., Böhm M., Schütt J., Pütz N., Baraban L., Eng L. M., Cuniberti G. (2018). Plasmonic Biosensor Based on Vertical Arrays of Gold
Nanoantennas. ACS Sens..

[3] Riley J. A., Horák M., Křápek V., Healy N., Pacheco-Peña V. (2023). Plasmonic
sensing using Babinet’s principle. Nanophotonics.

[4] Yang Y., Jia H., Hu N., Zhao M., Li J., Ni W., Zhang C.-y. (2024). Construction
of Gold/Rhodium Freestanding Superstructures
as Antenna-Reactor Photocatalysts for Plasmon-Driven Nitrogen Fixation. J. Am. Chem. Soc..

[5] Ni X., Ishii S., Kildishev A. V., Shalaev V. M. (2013). Ultra-thin, planar,
Babinet-inverted plasmonic metalenses. Light:
Sci. Appl..

[6] Rovenská K., Ligmajer F., Idesová B., Kepič P., Liška J., Chochol J., Šikola T. (2023). Structural
color filters with compensated angle-dependent shifts. Opt. Express.

[7] Zorić I., Zäch M., Kasemo B., Langhammer C. (2011). Gold, Platinum,
and Aluminum Nanodisk Plasmons: Material Independence, Subradiance,
and Damping Mechanisms. ACS Nano.

[8] Horák M., Čalkovský V., Mach J., Křápek V., Šikola T. (2023). Plasmonic
Properties of Individual Gallium Nanoparticles. J. Phys. Chem. Lett..

[9] Hopper E. R., Boukouvala C., Asselin J., Biggins J. S., Ringe E. (2022). Opportunities
and Challenges for Alternative Nanoplasmonic Metals: Magnesium and
Beyond. J. Phys. Chem. C.

[10] Gao Z., Wildenborg A., Kocoj C. A., Liu E., Sheofsky C., Rawashdeh A., Qu H., Guo P., Suh J. Y., Yang A. (2023). Low-Loss Plasmonics
with Nanostructured Potassium and Sodium–Potassium
Liquid Alloys. Nano Lett..

[11] Knight M. W., King N. S., Liu L., Everitt H. O., Nordlander P., Halas N. J. (2014). Aluminum for Plasmonics. ACS
Nano.

[12] Ligmajer F., Horák M., Šikola T., Fojta M., Daňhel A. (2019). Silver Amalgam
Nanoparticles and Microparticles: A Novel Plasmonic Platform for Spectroelectrochemistry. J. Phys. Chem. C.

[13] Liao Y., Fan Y., Lei D. (2024). Thermally tunable binary-phase
VO2 metasurfaces for
switchable holography and digital encryption. Nanophotonics.

[14] Kepič P., Horák M., Kabát J., Hájek M., Konečná A., Šikola T., Ligmajer F. (2025). Coexisting Phases of Individual VO2 Nanoparticles for
Multilevel Nanoscale Memory. ACS Nano.

[15] McMahon J. M., Schatz G. C., Gray S. K. (2013). Plasmonics
in the ultraviolet with
the poor metals Al, Ga, In, Sn, Tl, Pb, and Bi. Phys. Chem. Chem. Phys..

[16] Behnia K., Méasson M.-A., Kopelevich Y. (2007). Nernst Effect in Semimetals: The
Effective Mass and the Figure of Merit. Phys.
Rev. Lett..

[17] Tian Y., Toudert J. (2018). Nanobismuth: Fabrication, Optical, and Plasmonic Properties—Emerging
Applications. J. Nanotechnol..

[18] Wang Y. W., Kim J. S., Kim G. H., Kim K. S. (2006). Quantum size effects
in the volume plasmon excitation of bismuth nanoparticles investigated
by electron energy loss spectroscopy. Appl.
Phys. Lett..

[19] Lee S., Ham J., Jeon K., Noh J.-S., Lee W. (2010). Direct observation
of the semimetal-to-semiconductor transition of individual single-crystal
bismuth nanowires grown by on-film formation of nanowires. Nanotechnology.

[20] Son J. S., Park K., Han M., Kang C., Park S., Kim J., Kim W., Kim S., Hyeon T. (2011). Large-Scale Synthesis
and Characterization of the Size-Dependent Thermoelectric Properties
of Uniformly Sized Bismuth Nanocrystals. Angew.
Chem., Int. Ed..

[21] Leng D., Wang T., Li Y., Huang Z., Wang H., Wan Y., Pei X., Wang J. (2021). Plasmonic Bismuth Nanoparticles:
Thiolate Pyrolysis Synthesis, Size-Dependent LSPR Property, and Their
Oxidation Behavior. Inorg. Chem..

[22] Martínez-Lara D., González-Campuzano R., Mendoza D. (2022). Bismuth plasmonics
in the visible spectrum using texturized films. Photonics Nanostruct. - Fundam. Appl..

[23] Toudert J., Serna R., de Castro M. J. (2012). Exploring
the Optical Potential of
Nano-Bismuth: Tunable Surface Plasmon Resonances in the Near Ultraviolet-to-Near
Infrared Range. J. Phys. Chem. C.

[24] Ozbay I., Ghobadi A., Butun B., Turhan-Sayan G. (2020). Bismuth plasmonics
for extraordinary light absorption in deep sub-wavelength geometries. Opt. Lett..

[25] Chacon-Sanchez F., de Galarreta C. R., Nieto-Pinero E., Garcia-Pardo M., Garcia-Tabares E., Ramos N., Castillo M., Lopez-Garcia M., Siegel J., Toudert J., Wright C. D., Serna R. (2024). Building Conventional
Metasurfaces with Unconventional Interband Plasmonics: A Versatile
Route for Sustainable Structural Color Generation Based on Bismuth. Adv. Opt. Mater..

[26] Tomaszewska E., Soliwoda K., Kadziola K., Tkacz-Szczesna B., Celichowski G., Cichomski M., Szmaja W., Grobelny J. (2013). Detection
Limits of DLS and UV-Vis Spectroscopy in Characterization of Polydisperse
Nanoparticles Colloids. J. Nanomater..

[27] Hendel T., Wuithschick M., Kettemann F., Birnbaum A., Rademann K., Polte J. (2014). In Situ Determination
of Colloidal Gold Concentrations with UV–Vis
Spectroscopy: Limitations and Perspectives. Anal. Chem..

[28] García
de Abajo F. J. (2010). Optical excitations in electron microscopy. Rev. Mod. Phys..

[29] Horák M., Bukvišová K., Švarc V., Jaskowiec J., Křápek V., Šikola T. (2018). Comparative
study of plasmonic antennas fabricated by electron beam and focused
ion beam lithography. Sci. Rep..

[30] Horák M., Šikola T. (2020). Influence of experimental conditions
on localized surface
plasmon resonances measurement by electron energy loss spectroscopy. Ultramicroscopy.

[31] Křápek V., Konečná A., Horák M., Ligmajer F., Stöger-Pollach M., Hrtoň M., Babocký J., Šikola T. (2020). Independent
engineering of individual
plasmon modes in plasmonic dimers with conductive and capacitive coupling. Nanophotonics.

[32] Sano Y., Satoh H., Chiba M., Shinohara A., Okamoto M., Serizawa K., Nakashima H., Omae K. (2005). A 13-Week Toxicity Study of Bismuth in Rats by Intratracheal Intermittent
Administration. J. Occup. Health.

[33] Griffith D. M., Li H., Werrett M. V., Andrews P. C., Sun H. (2021). Medicinal chemistry
and biomedical applications of bismuth-based compounds and nanoparticles. Chem. Soc. Rev..

[34] Waxenegger J., Trügler A., Hohenester U. (2015). Plasmonics simulations with the MNPBEM
toolbox: Consideration of substrates and layer structures. Comput. Phys. Commun..

[35] Werner W. S. M., Glantschnig K., Ambrosch-Draxl C. (2009). Optical Constants
and Inelastic Electron-Scattering
Data for 17 Elemental Metals. J. Phys. Chem.
Ref. Data.

[36] Foltýn, M. ; Šikola, T. ; Horák, M. Bismuth Plasmonic Antennas. 2025, arXiv:2504.00671. arXiv.org e-Printarchive. https://arxiv.org/abs/2504.00671. (accessed: August 24, 2025). 10.1021/acsnano.5c07482 PMC12444988 40888192

