# Supplementary material for: Bismuth Plasmonic Antennas
Source: ACS Nano. 2025 Sep 1;19(36):32299–305. doi: 10.1021/acsnano.5c07482 (PMC12444988; doi:10.1021/acsnano.5c07482)
Supplement: Supplementary file 1 [file nn5c07482_si_001.pdf]

# Supporting Information: Bismuth Plasmonic Antennas

Michael Foltýn,[†] Tomáš Šikola,[†,‡] and Michal Horák[*,†]

†*Brno University of Technology, Central European Institute of Technology, Purkyňova 123, Brno, 612 00, Czech Republic*

‡*Brno University of Technology, Faculty of Mechanical Engineering, Institute of Physical Engineering, Technická 2, Brno, 616 69, Czech Republic*

E-mail: michal.horak2@ceitec.vutbr.cz

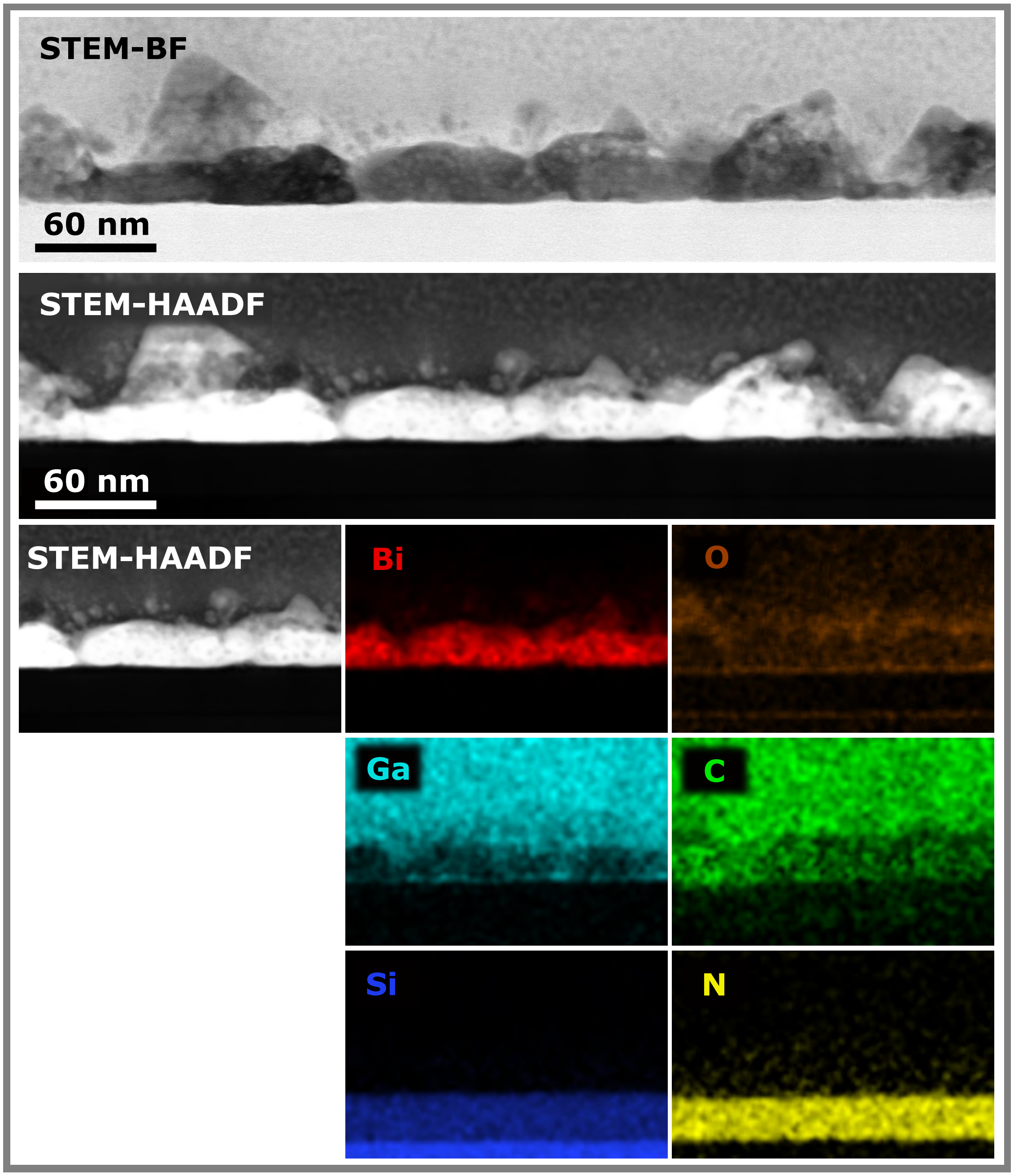


Figure S1: Material analysis of the 30 nm thick bismuth thin film deposited on a 30 nm thick silicon nitride membrane performed by STEM EDX (energy-dispersive X-ray spectroscopy) mapping of Bi, O, Ga, C, Si, and N. The cross-sectional view shows the pronounced roughness of the bismuth layer. The tiny particles above the bismuth layer can be attributed either to the redeposition of bismuth during the lamella fabrication or to residuals of not fully imaged grains. The composition is from top to bottom the following: lamella protection layer (carbon and gallium), bismuth layer, silicon nitride membrane, and silicon supportive frame. The presence of oxygen can be attributed to the plasma cleaning of the lamella in argon-oxygen plasma before the STEM EDX analysis to prevent contamination.

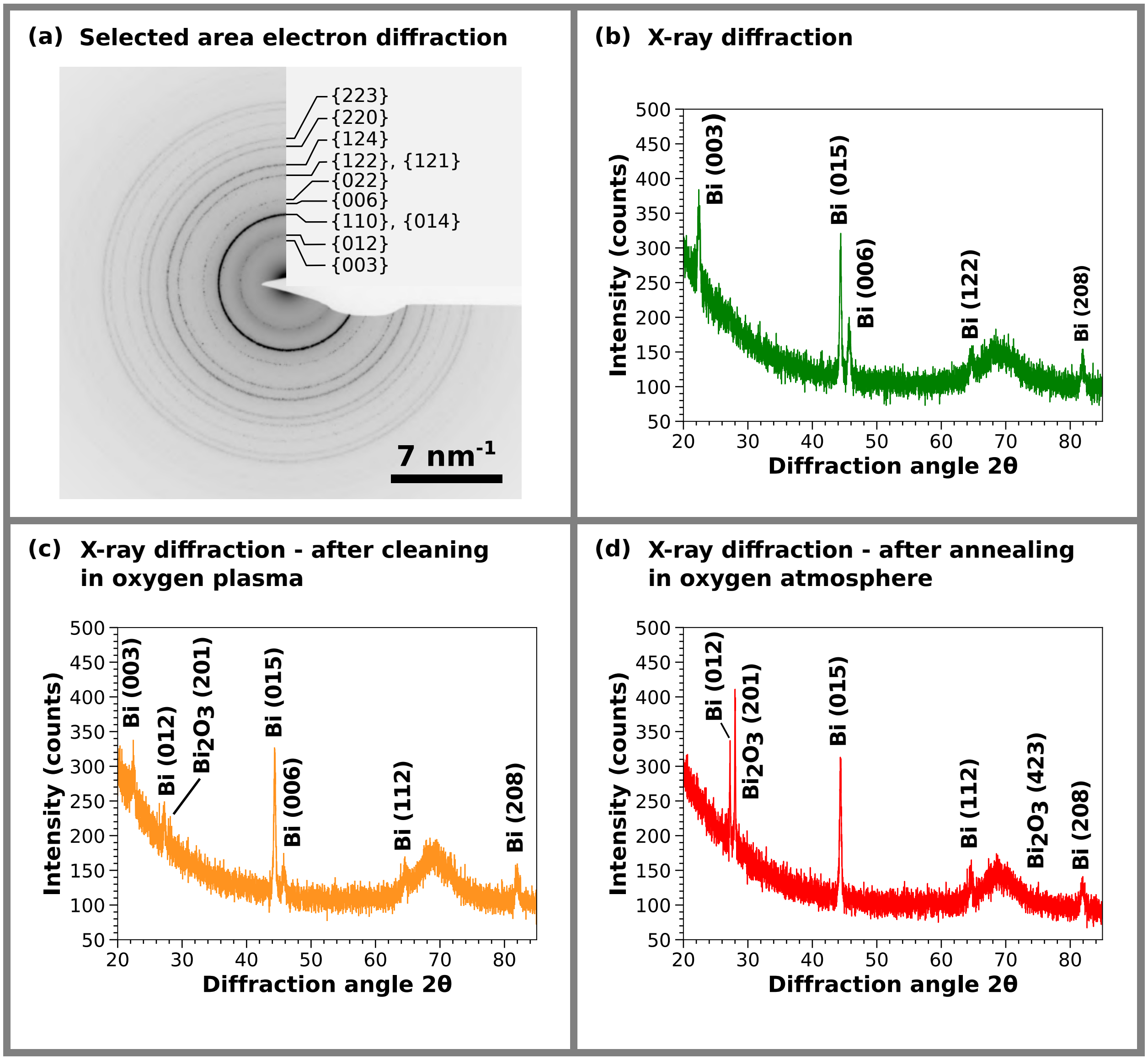


Figure S2: Diffraction analysis of the 30 nm thick bismuth thin film deposited on a 30 nm thick silicon nitride membrane performed (a) by selective area electron diffraction (SAED) in the TEM and (b) by X-ray diffraction (XRD). The XRD pattern of the bismuth layer contains 5 peaks corresponding to bismuth, namely Bi(003), Bi(015), Bi(006), Bi(122), and Bi(208), and a broad peak corresponding to the silicon nitride membrane. Both diffractograms contain no signs of the presence of bismuth oxide, therefore the bismuth layer is not oxidized. Panel (c) shows the XRD pattern of bismuth layer that was plasma cleaned in oxygen plasma. As a result, Bi(003) peak decreases, and two additional peaks have appeared: Bi(012) and $Bi_2O_3$(201). This is a sign of surface oxidation by oxygen plasma. Finally, panel (d) shows the XRD pattern of bismuth layer that was annealed in an oxygen atmosphere, which resulted in the vanishing of the Bi(003) and Bi(006) peaks, an increase of Bi(012) and $Bi_2O_3$(201) peaks, and additional bismuth oxide peak $Bi_2O_3$(423) appeared. This is a sign of further oxidation of bismuth by the annealing in an oxygen atmosphere.

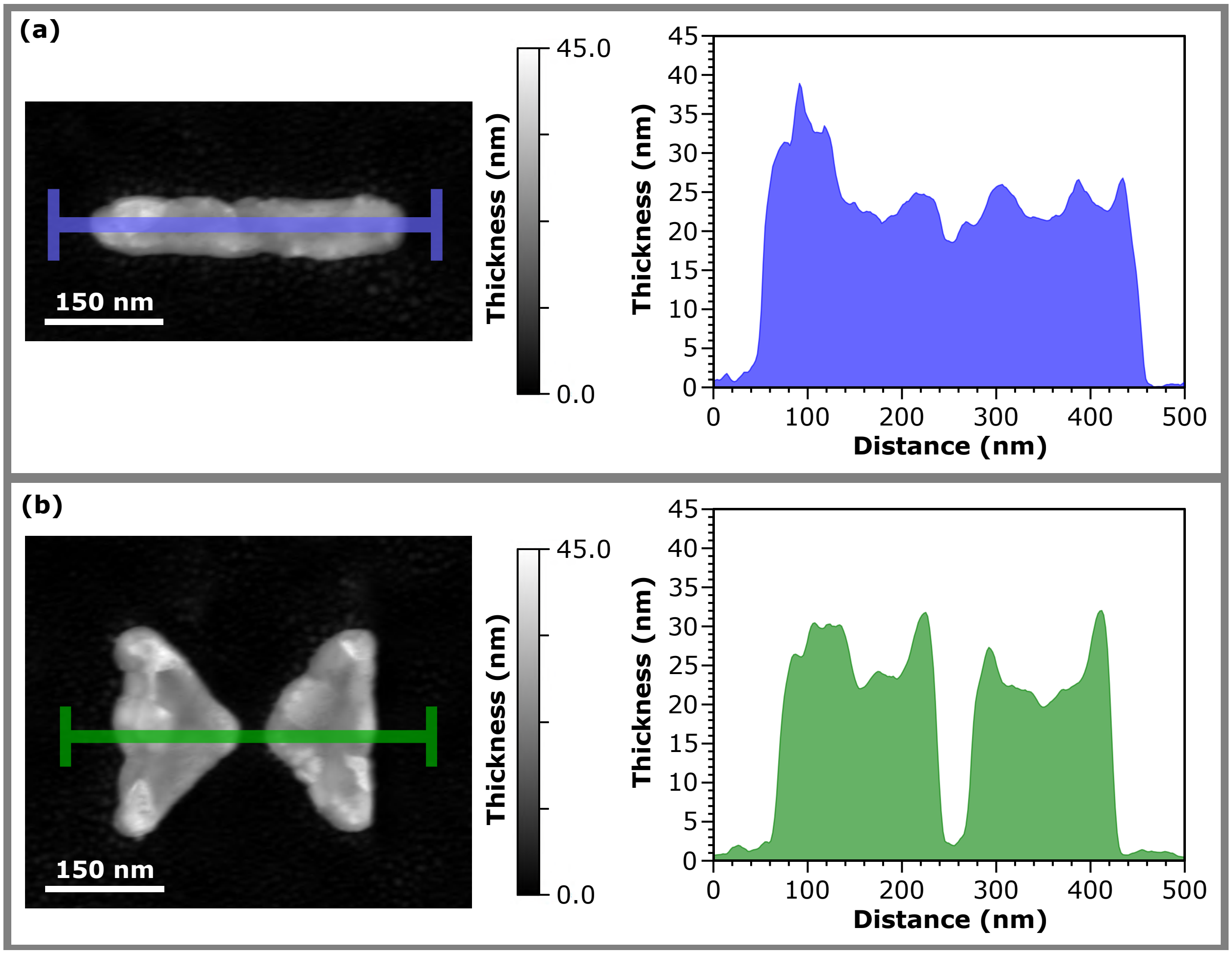


Figure S3: Thickness maps and thickness profiles of (a) a 410 nm long bar-shaped and (b) a 288 nm wide bowtie bismuth antenna measured by STEM EELS. The nominal thickness of the bismuth thin film was 30 nm. The relative thickness was evaluated using the log-ratio method. The absolute thickness was calculated using the inelastic mean free path of 120 keV electrons in bismuth equal to 105 nm.

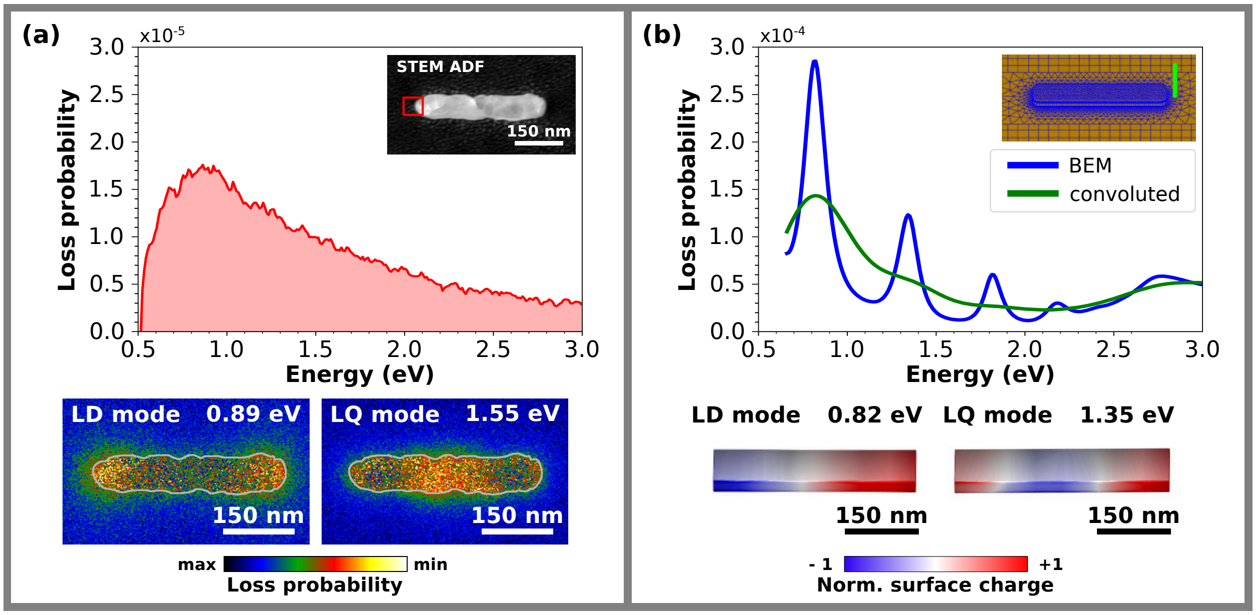


Figure S4: Plasmonic response of a 410 nm long bar-shaped bismuth antenna. (a) STEM ADF micrograph of the bar, EEL spectrum, and energy filtered loss probability maps at the energy of 0.89 eV and 1.55 eV corresponding to the longitudinal dipole (LD) and longitudinal quadrupole (LQ) mode. (b) Numerical simulation of the EEL specrtum that is further convolved with a Gaussian function to reproduce the instrumental broadening of the peaks. The LD and LQ mode is visualized by the normalized surface charge distributions at the peak energy of these modes (0.82 eV and 1.35 eV). The theory represented by numerical simulations matches reasonably the experiment.

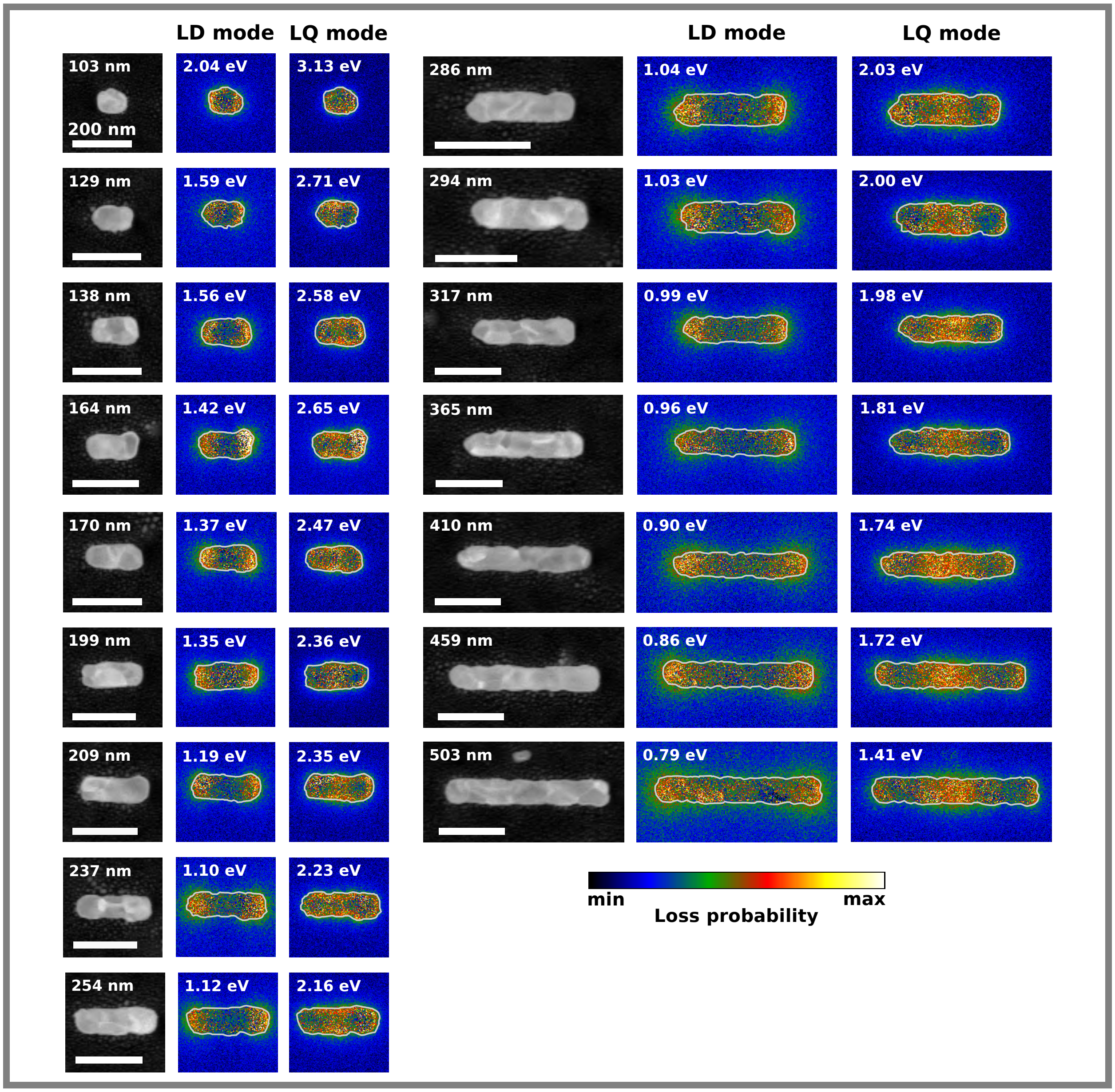


Figure S5: Energy filtered loss probability maps of the bar-shaped bismuth antennas at the energies corresponding to their longitudinal dipole (LD) and longitudinal quadrupole (LQ) modes. The scalebars are 200 nm long.

Table S1: Parameters of LD and LQ mode in the bar antennas – the energy of the respective LSPR mode $E$, the loss probability maximum, and the FWHM of the peak $\Delta E$.

| size | LD mode | | | LQ mode | | |
|---|---|---|---|---|---|---|
| (nm) | $E$ (eV) | loss prob. | $\Delta E$ (eV) | $E$ (eV) | loss prob. | $\Delta E$ (eV) |
| 103 | 2.04 | $1.3 \cdot 10^{-5}$ | 0.65 | 3.13 | $5.1 \cdot 10^{-6}$ | 0.36 |
| 129 | 1.59 | $1.5 \cdot 10^{-5}$ | 0.50 | 2.71 | $5.2 \cdot 10^{-6}$ | 0.34 |
| 138 | 1.56 | $1.5 \cdot 10^{-5}$ | 0.49 | 2.58 | $5.6 \cdot 10^{-6}$ | 0.33 |
| 164 | 1.42 | $1.8 \cdot 10^{-5}$ | 0.44 | 2.65 | $9.7 \cdot 10^{-6}$ | 0.46 |
| 170 | 1.37 | $1.8 \cdot 10^{-5}$ | 0.43 | 2.47 | $7.7 \cdot 10^{-6}$ | 0.43 |
| 199 | 1.35 | $2.3 \cdot 10^{-5}$ | 0.44 | 2.36 | $9.2 \cdot 10^{-6}$ | 0.30 |
| 209 | 1.19 | $1.9 \cdot 10^{-5}$ | 0.42 | 2.35 | $1.1 \cdot 10^{-5}$ | 0.45 |
| 237 | 1.10 | $1.7 \cdot 10^{-5}$ | 0.43 | 2.23 | $8.2 \cdot 10^{-6}$ | 0.45 |
| 254 | 1.12 | $1.9 \cdot 10^{-5}$ | 0.37 | 2.16 | $8.1 \cdot 10^{-6}$ | 0.43 |
| 286 | 1.04 | $2.1 \cdot 10^{-5}$ | 0.36 | 2.03 | $9.9 \cdot 10^{-6}$ | 0.42 |
| 294 | 1.03 | $2.2 \cdot 10^{-5}$ | 0.38 | 2.00 | $9.7 \cdot 10^{-6}$ | 0.35 |
| 317 | 0.99 | $2.0 \cdot 10^{-5}$ | 0.37 | 1.98 | $8.7 \cdot 10^{-6}$ | 0.39 |
| 365 | 0.95 | $2.0 \cdot 10^{-5}$ | 0.37 | 1.81 | $8.1 \cdot 10^{-6}$ | 0.29 |
| 410 | 0.90 | $1.7 \cdot 10^{-5}$ | 0.36 | 1.74 | $7.1 \cdot 10^{-6}$ | 0.32 |
| 459 | 0.85 | $2.1 \cdot 10^{-5}$ | 0.36 | 1.72 | $1.0 \cdot 10^{-5}$ | 0.33 |
| 503 | 0.80 | $2.2 \cdot 10^{-5}$ | 0.22 | 1.41 | $1.4 \cdot 10^{-5}$ | 0.37 |

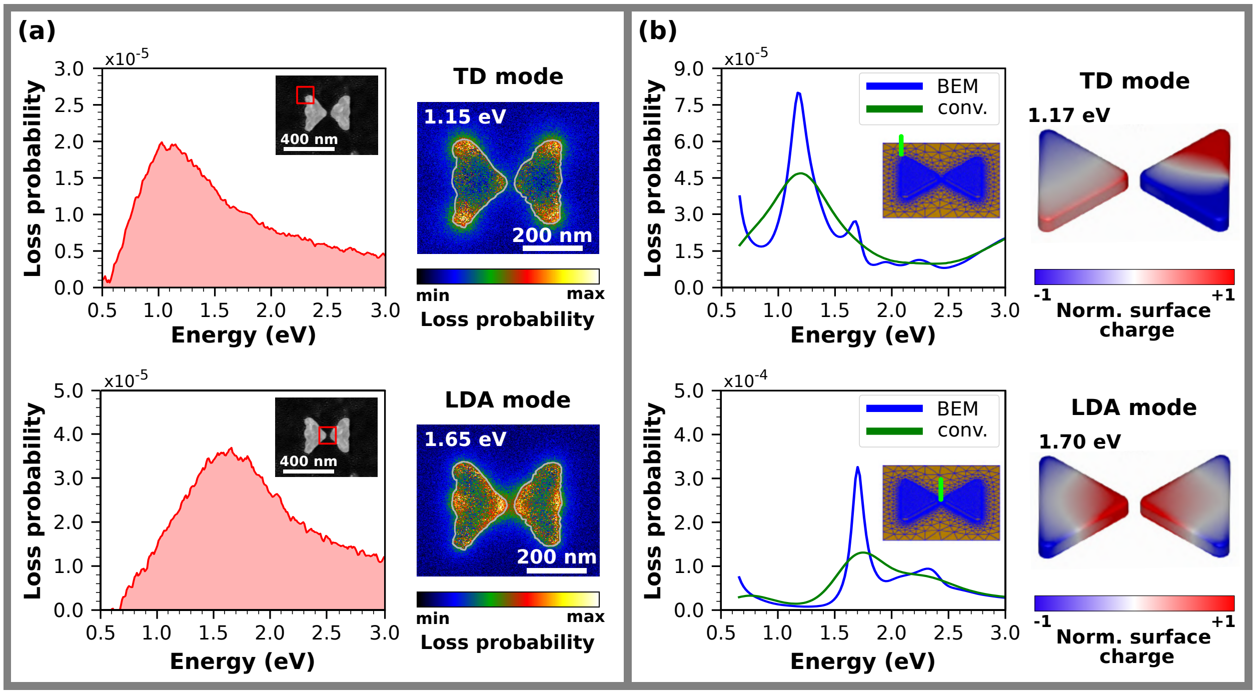


Figure S6: Plasmonic response of a 288 nm wide bowtie bismuth antenna. (a) STEM ADF micrograph of the bowtie, EEL spectra recorded at the outer corner and in the gap of the bowtie, and energy filtered loss probability maps at the energy of 1.15 eV and 1.65 eV corresponding to the transverse dipole (TD) and longitudinal dipole antibonding (LDA) mode, respectively. (b) Numerical simulations of the EEL spectra at the outer corner and in the gap of the bowtie that are further convolved with a Gaussian function to reproduce the instrumental broadening of the peaks. The TD and LDA mode is visualized by the normalized surface charge distributions at the peak energy of these modes (1.17 eV and 1.70 eV). The theory represented by numerical simulations matches well the experiment.

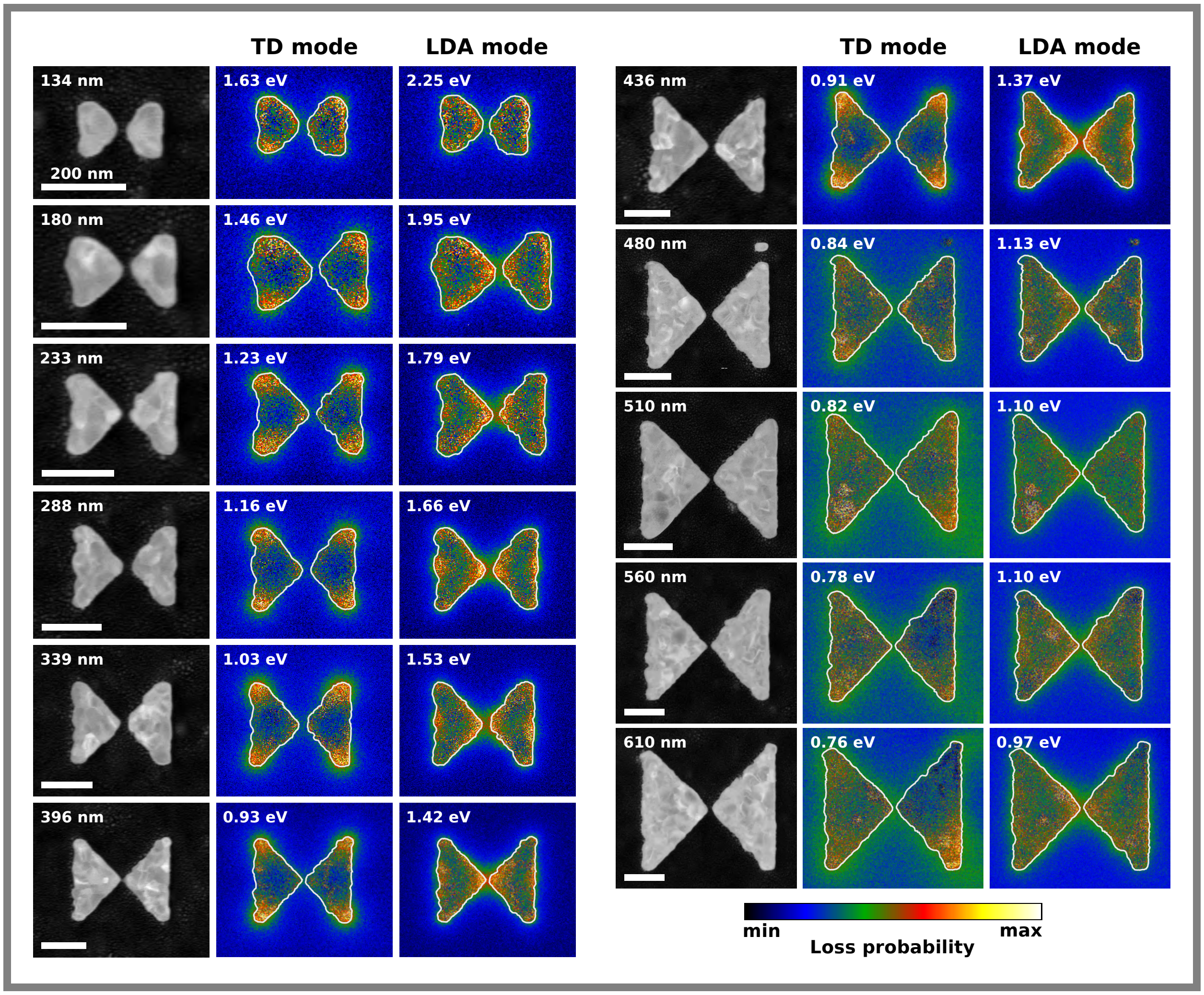


Figure S7: Energy filtered loss probability maps of the bowtie bismuth antennas at the energies corresponding to their transverse dipole (TD) and longitudinal dipole antibonding (LDA) modes. The scalebars are 200 nm long.

Table S2: Parameters of TD and LDA mode in the bowtie antennas – the energy of the respective LSPR mode $E$, the loss probability maximum, and the FWHM of the peak $\Delta E$.

| size | TD mode | | | LDA mode | | |
|---|---|---|---|---|---|---|
| (nm) | $E$ (eV) | loss prob. | $\Delta E$ (eV) | $E$ (eV) | loss prob. | $\Delta E$ (eV) |
| 134 | 1.63 | $1.5 \cdot 10^{-5}$ | 0.54 | 2.25 | $1.4 \cdot 10^{-5}$ | 0.40 |
| 180 | 1.46 | $1.7 \cdot 10^{-5}$ | 0.56 | 1.95 | $3.0 \cdot 10^{-5}$ | 0.50 |
| 233 | 1.23 | $2.0 \cdot 10^{-5}$ | 0.36 | 1.79 | $3.2 \cdot 10^{-5}$ | 0.50 |
| 288 | 1.16 | $2.0 \cdot 10^{-5}$ | 0.34 | 1.66 | $3.6 \cdot 10^{-5}$ | 0.49 |
| 339 | 1.03 | $2.2 \cdot 10^{-5}$ | 0.31 | 1.53 | $3.8 \cdot 10^{-5}$ | 0.49 |
| 396 | 0.93 | $2.6 \cdot 10^{-5}$ | 0.31 | 1.42 | $4.4 \cdot 10^{-5}$ | 0.47 |
| 436 | 0.91 | $2.4 \cdot 10^{-5}$ | 0.31 | 1.37 | $4.2 \cdot 10^{-5}$ | 0.42 |
| 480 | 0.84 | $1.1 \cdot 10^{-5}$ | 0.30 | 1.13 | $3.0 \cdot 10^{-5}$ | 0.32 |
| 510 | 0.82 | $1.7 \cdot 10^{-5}$ | 0.23 | 1.10 | $2.9 \cdot 10^{-5}$ | 0.33 |
| 560 | 0.78 | $1.2 \cdot 10^{-5}$ | 0.20 | 1.10 | $3.5 \cdot 10^{-5}$ | 0.33 |
| 620 | 0.76 | $1.5 \cdot 10^{-5}$ | 0.17 | 0.97 | $3.9 \cdot 10^{-5}$ | 0.25 |

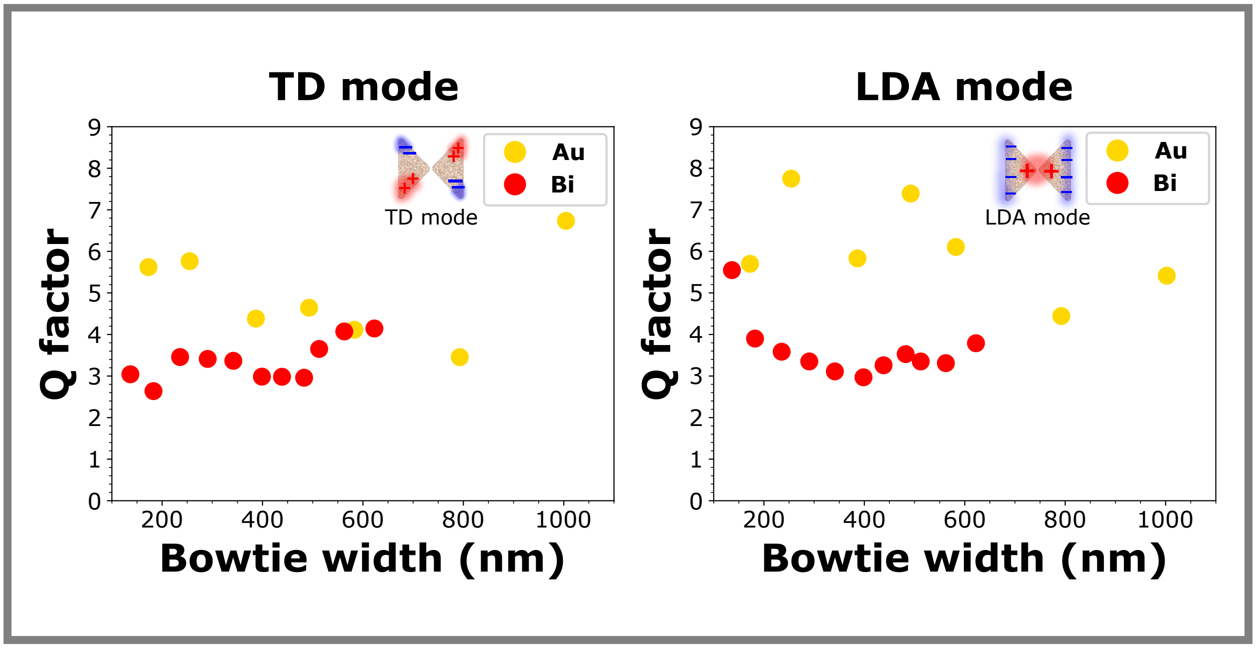


Figure S8: Comparison of the Q factors for the TD and LDA modes in the bowtie bismuth and gold antennas.
